# Doppler is a Safe Criterion for Ensuring the Implementation of Eversion Carotid Endarterectomy

**DOI:** 10.3400/avd.oa.21-00065

**Published:** 2021-12-25

**Authors:** Panagitsa D. Christoforou, Chris N. Bakoyiannis, Marianna Konidari, Sotirios Georgopoulos, Thomas Kotsis

**Affiliations:** 1 Vascular Department, 2nd Clinic of Surgery, Aretaieion University Hospital, Athens Medical School, National and Kapodistrian University of Athens, Athens, Greece; 2 Vascular Department, 1st Clinic of Surgery, Laiko General Hospital, Athens Medical School, National and Kapodistrian University of Athens, Athens, Greece; 3 1st Department of Radiology, Aretaieion University Hospital, Athens Medical School, National and Kapodistrian University of Athens, Athens, Greece

**Keywords:** carotid stenosis, eversion carotid endarterectomy, internal carotid artery, peripheral extension, Doppler study

## Abstract

**Objective:** This is a prospective study concerning patients with symptomatic or asymptomatic significant carotid stenosis. Preoperative and intraoperative measurements of the peripheral extension of the carotid atherosclerotic plaque have been compared with postoperative measurements to identify a preoperative method that safely allows the performance of eversion carotid endarterectomy (ECEA).

**Materials and Methods:** The study included 37 patients with symptomatic internal carotid stenosis greater than 70% and 43 patients with asymptomatic stenosis greater than 80%. Four methods were used for establishing criteria: preoperative Doppler examination, intraoperative measurement of the carotid atheroma before artery division, measurement of the removed plaque, and histological measurement of the plaque.

**Results:** Preoperative Doppler examination is a method of estimating the actual distal extension of the internal carotid artery (ICA) atheroma, with correction as dictated by the following formula:

AL= 0.6704+0.7685⋅Doppler

In all cases, preoperative ultrasound measurements and intraoperative estimation confirmed the correct decision to proceed with the eversion technique.

**Conclusion:** The peripheral extension of the atherosclerotic plaque of the ICA can be assessed with accuracy by preoperative Doppler study, which can be used with safety as a predicting criterion of the existence of healthy peripheral carotid tissue that allows the performance of the eversion endarterectomy technique.

## Introduction

Several historical studies, such as the North American Symptomatic Carotid Endarterectomy Trial (NASCET) and the Asymptomatic Carotid Atherosclerosis Study (ACAS), document the need to treat carotid stenotic disease as a prevention of a new ischemic stroke.

According to the latest European Society for Vascular Surgery (ESVS) guidelines, eversion carotid endarterectomy (ECEA) (ESVS Recommendation 55-1A) is a surgical technique that now appears to be an effective and alternative method of the traditional carotid endarterectomy with patch^1)^; ECEA offers many advantages, such as the ellipsoid anastomosis, correction of a tortuous dolicho-mega carotid artery, and avoidance of a patch.^2)^ However, this technique still lies in the shadow of the longitudinal carotid endarterectomy mainly for one reason: the possible distal extension of the atherosclerotic plaque beyond classical surgical borders, up to the skull base. The technique of tacking sutures, a common method of fixing the residual distal atheroma in the classical carotid endarterectomy, is a more complicated procedure in an already divided, everted internal carotid; thus, before choosing the method of eversion, it is imperative to know with a high degree of certainty the distal extension of the atheroma. In the case of a prohibited peripheral extension, one could choose the traditional endarterectomy, before the division of the carotid, which is the point of almost no return considering the eversion method.

We conducted a prospective study aimed at assessing the degree of certainty of the preoperative paraclinical studies and the intraoperative estimation of the atheroma distal extension, before the carotid division, in comparison with the actual length (AL) of the atheromatic plaque. If there is a reliable method that safely estimates the peripheral level of the plaque, this can be used as a preoperative criterion to perform the eversion technique or use the traditional carotid endarterectomy (e.g., patch endarterectomy).

## Materials and Methods

In the Vascular Unit of the 2nd Clinic of Surgery of the Aretaieion University Hospital of Athens, 82 carotid endarterectomies were performed under general anesthesia by the same surgeon (Prof. Thomas Kotsis) in a period of 36 months.

### Selection criteria

In 80 cases, the patients underwent ECEA to treat extracranial vascular disease. As for the two other cases, in one patient’s case, a linear cross section with primary closure was performed; in the other patient, with an elongated cross section, a synthetic patch was used after carotid restenosis due to a previous endarterectomy 14 years ago. At the same period, in two symptomatic patients with concomitant heart disease, it was deemed necessary to simultaneously treat carotid stenosis and coronary artery stenosis in another hospital with the simultaneous presence of a cardiac and a vascular surgeon.

The exclusion criteria from the surgery were the simultaneous presence of intracranial internal carotid artery (ICA) stenosis greater than the degree of the ipsilateral ICA or obstruction of intracranial cerebral vessels according to brain imaging evaluation with computed tomography angiography (CTA), magnetic resonance angiography (MRA), or digital subtraction angiography (DSA), as well a severe disability according to the modified Rankin scale with a score above 4. The patients included in the study underwent ECEA without patient selection, except for the two above exceptions.

### Patients

The study included 37 patients with symptomatic ICA stenosis greater than 70% and 43 patients with asymptomatic stenosis greater than 80%, based on the criteria for NASCET carotid stenosis measurement.^3)^ Only 25% of the patients had ICA stenosis of 70%–79%, while 40% and 35% of the patients had ICA stenosis of 80%–89% and 90%–99%, respectively.

Most patients (53.75%) were under 70 years old, while 29 (36.25%) were between 70 and 79 years old. Of the patients, 10% were 80 years old or older. The mean age of the patients was 70.1±7.5 years.

The patients were divided into two groups according to symptomatology: those who were symptomatic, which included 10 female and 27 male, and those who were asymptomatic, while included 16 female and 27 male. The mean age of the female and male patients was 69.2±8.86 and 70.5±6.8 years, respectively.

The aim of this perioperative study on imaging and measurements, which include the categories preoperative, intraoperative, and postoperative studies and measurements, is to determine the peripheral extension of the atheroma ICA. We then analyzed each category separately.

#### Preoperative studies and measurements

All patients underwent preoperative, diagnostic imaging performed by the same radiologist, with the presence of a vascular surgeon. The procedure included a Duplex color ultrasound using the GE LOGIQ9 ultrasound system (General Electric, New York, NY, USA). Sixty-nine patients underwent DSA using the Philips Allura Xper FD20 angiography system (PHILIPS, Amsterdam, Netherlands), nine underwent CTA, and two underwent MRA. All procedures were completed at the Aretaieion University Hospital of Athens.

The color Duplex ultrasound study provided information related to the atherosclerotic plaque of the ICA, which is used in the study to assess the extension both peripherally and centrally and calculate the degree of stenosis with hemodynamic and morphological parameters. To determine the degree of stenosis ultrasonographically, the diagnostic velocity criteria for NASCET-based carotid stenosis measurement were used as reference levels, which specified that maximum systolic velocity values (peak systolic velocity) greater than 230 cm/s and end-diastolic velocity values (end-diastolic velocity) greater than 100 cm/s indicate stenosis greater than 70%.

For statistical analysis, the measurement of atherosclerotic plaque during the preoperative ultrasound study was indicated as the Doppler measurement length (DML).

Intra-arterial DSA of the aortic arch and its branches was performed to confirm the degree of carotid stenosis; it provides information on the nature and extent of atherosclerotic plaque and the patency of the ipsilateral common carotid artery (CCA) and unilateral ICA. The angiographic examination also provides important information regarding the existence of an adequate Willis’ circle, as well as valuable details related to the planning of the vascular surgery.

#### Intraoperative studies and measurements

In addition to preoperative measurements and observations, intraoperative measurements, through the surgeon’s senses, are also important in assessing peripheral plaque extension.

Through bare eye inspection, an opaque formation is sometimes identified on the outer surface in the area of the stenosis, corresponding to the altered color of the vessel due to the attack by the atherosclerotic plaque. The peripheral transition to the normal color of the vessel is usually obvious and is an important detail because it presupposes the determination of the distal expansion of the atherosclerotic plaque. In this way, the level of the peripheral placement of the clamp can be estimated at a distance that allows surgical manipulations such as the reversal of the carotid wall and noncomplicated removal of the atheroma. On palpation, a hard surface is identified, and its distal end may correspond to the peripheral extension of the atheroma. Also, the inability to compress the ICA indicates the continuation of the atherosclerotic plaque. In our study, outside the vessel and before the oblique division of the internal carotid at the level of the carotid bifurcation, the length from the palpable atherosclerotic plaque to its estimated distal end was measured using a sterile ruler. For statistical analysis, the measurement of atherosclerotic plaque from the carotid bifurcation to the supposed distal end before the division was indicated as SURG.

**Figure 1:**
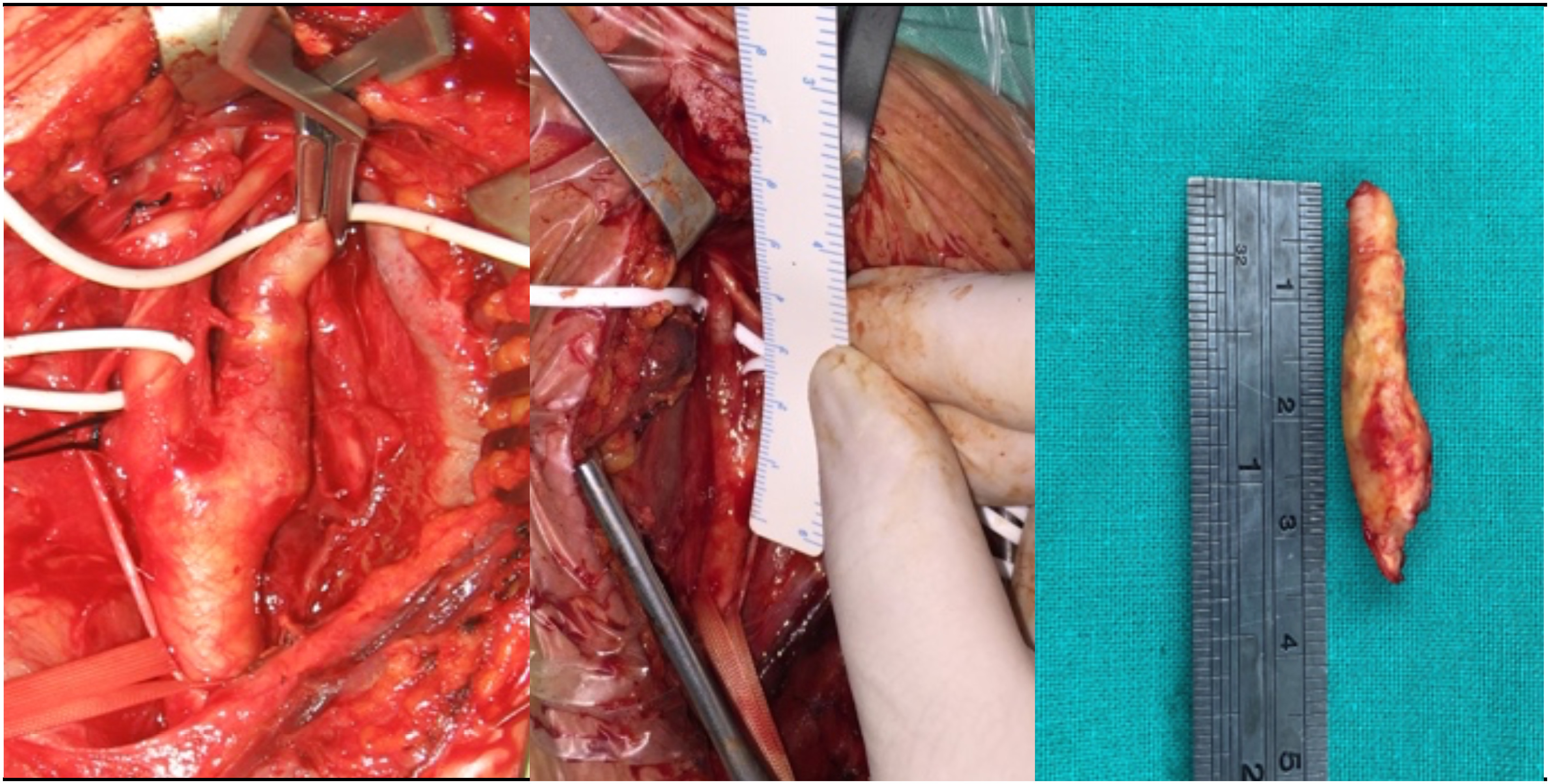
Fig. 1 Intraoperative findings during eversion carotid endarterectomy. An opaque formation is sometimes recognized on the outer surface, in the area of the stenosis corresponding to the color of the vessel, due to the development of the atherosclerotic plaque. The length of the palpable atherosclerotic plaque is measured and recorded on the outside of the vessel, before the oblique incision, using a sterile ruler. The length of the atheroma was measured after its removal from the internal carotid artery.

Immediately after the removal of the atheroma of the ICA, its length was measured on the back table, as well as the atheroma of the common carotid artery. For statistical analysis, the measurement of the atherosclerotic plaque of the ICA after its removal was indicated as the AL (**Fig. 1**).

#### Postoperative studies and measurements

Two pieces of the removed plaque (of the internal and common carotid artery) were sent for histopathological measurement and examination. For statistical analysis, the histopathological measurement of the atherosclerotic plaque of the ICA was indicated as HISTO.

**Figure 2:**
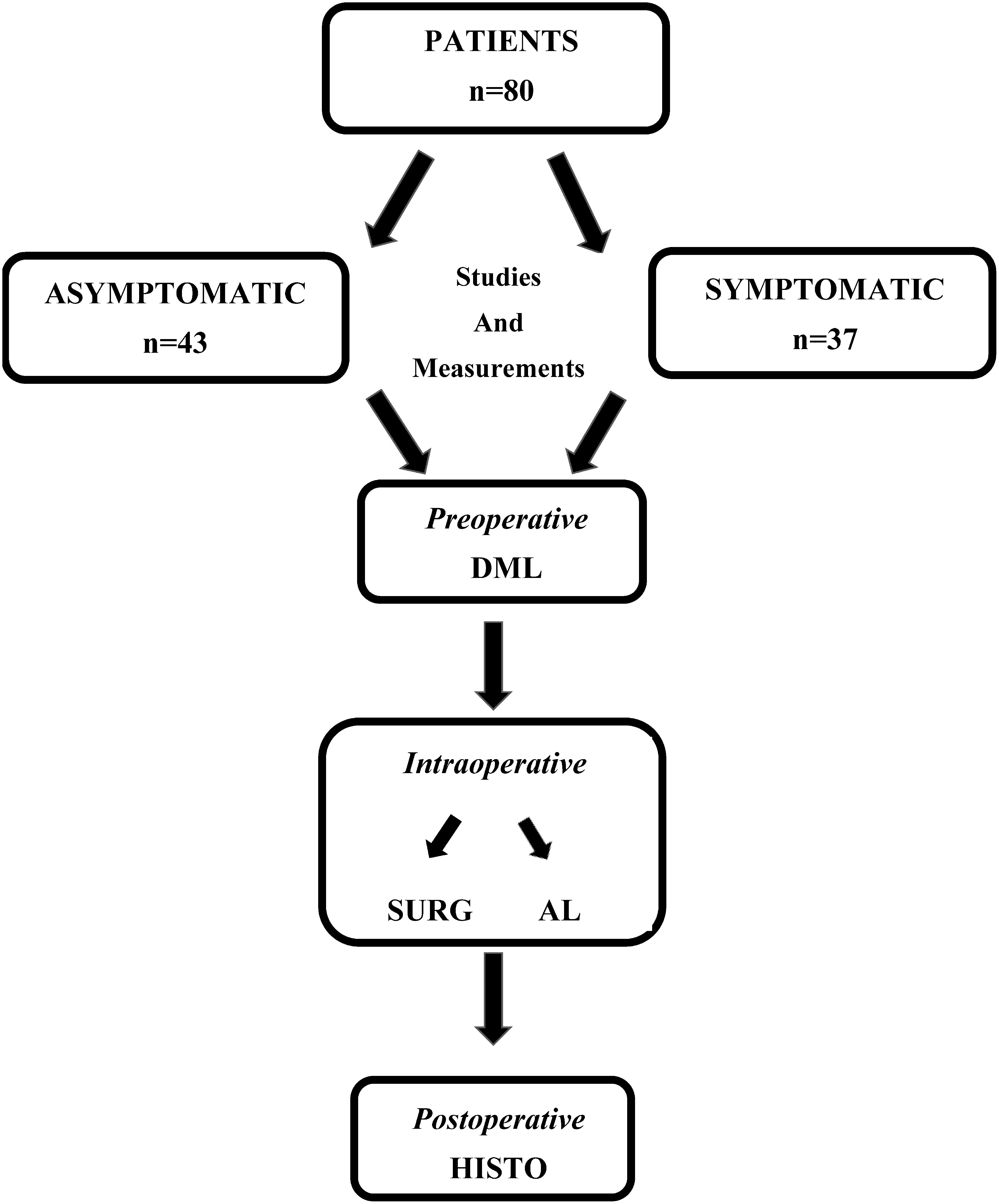
Fig. 2 Clinical trial flowchart describing the sequence and measurements of the study.

A clinical trial flowchart describing the sequence and measurements of the study was developed (**Fig. 2**). The preoperative, intraoperative, and postoperative studies and measurements of ICA atheromatic plaque were compared with each other to draw reliable conclusions on the distal extension of atheroma to proceed safely with the eversion carotid technique.

### Statistical analysis

The statistical analysis aimed to identify the relation of the preoperative DML of the atheromatic plaque to its AL as measured by the vascular surgeon using an appropriate measuring device (ruler) during the operation after its removal. It is important to ascertain if the DML is close enough to the AL so that the eversion technique can be safely performed. Additionally, two other measurement methods are investigated: a simple intraoperative measurement using the ruler before the removal of the atheromatic plaque from the carotid bifurcation to the supposed distal end before the division (SURG) and postoperative histological examination (HISTO). The data are presented as mean±standard deviation, minimum and maximum (continuous variables), and tables of frequencies (categorical variables) in the following tables.

To compare the three measurement methods (DML, SURG, and HISTO) to AL, the absolute differences from the AL measurement were computed and compared to the value of zero using Student’s t-test, the sign test, and Wilcoxon’s signed-rank test.

Furthermore, the potential impact of a set of demographic (gender) and risk factors [diabetes mellitus, smoking, hyperlipidemia, hypertension, previous coronary artery bypass grafting (CABG), percutaneous coronary intervention with stent (PCI-STENT), and peripheral artery disease (PAD)] was investigated, comparing the differences in the measurement of the various methods across the groups, defined by the presence or absence of the risk factor, and across gender. The analysis method used were Student’s t-test and Wilcoxon’s rank-sum test.

Finally, linear regression was used to quantify the relation between the AL and the DML. The objective was to estimate a model that could be used to correct/match the preoperative Doppler measurement, so that it could be a better approximation of the true length, thus facilitating surgical decision-making as far as the feasibility of the eversion technique, i.e., the length of the atheromatic plaque is within the limits allowing the operation to be a realistic option.

## Results

Τhe results of the statistical analysis^4–6)^ concerning the demographics and risk factors of the patients (**Table 1**), atheromatic plaque measurements by all four methods, and absolute differences in atheromatic plaque measurement from the AL method are presented below.

**Table 1:** Table 1 Demographics and risk factors of the patients

		Frequency	Percent		Frequency	Percent
Gender	Female	26	32.50	Hypertension	67	83.75
	Male	54	67.50	Diabetes	28	35.00
Location	Left	39	48.75	Smoke	43	53.75
	Right	41	51.25	Dyslipidemia	73	92.41
				CABG	10	12.66
				PCI-STENT	10	12.66
				PAD	21	26.58

CABG: coronary artery bypass grafting; PCI: percutaneous coronary intervention; PAD: peripheral artery disease

It should be noted that all three difference measures fail the test for normality (both Kolmogorov–Smirnov and Shapiro–Wilk tests), with p-value <0.01.

The absolute differences in atheromatic plaque measurement from the AL method, by gender and risk factors indicate that the DML difference is not affected by any of the auxiliary variables examined.

The regression analysis tested the following two models:

1. Simple linear regression of the AL measurement (dependent variable) on the DML (independent variable)

**Figure 3:**
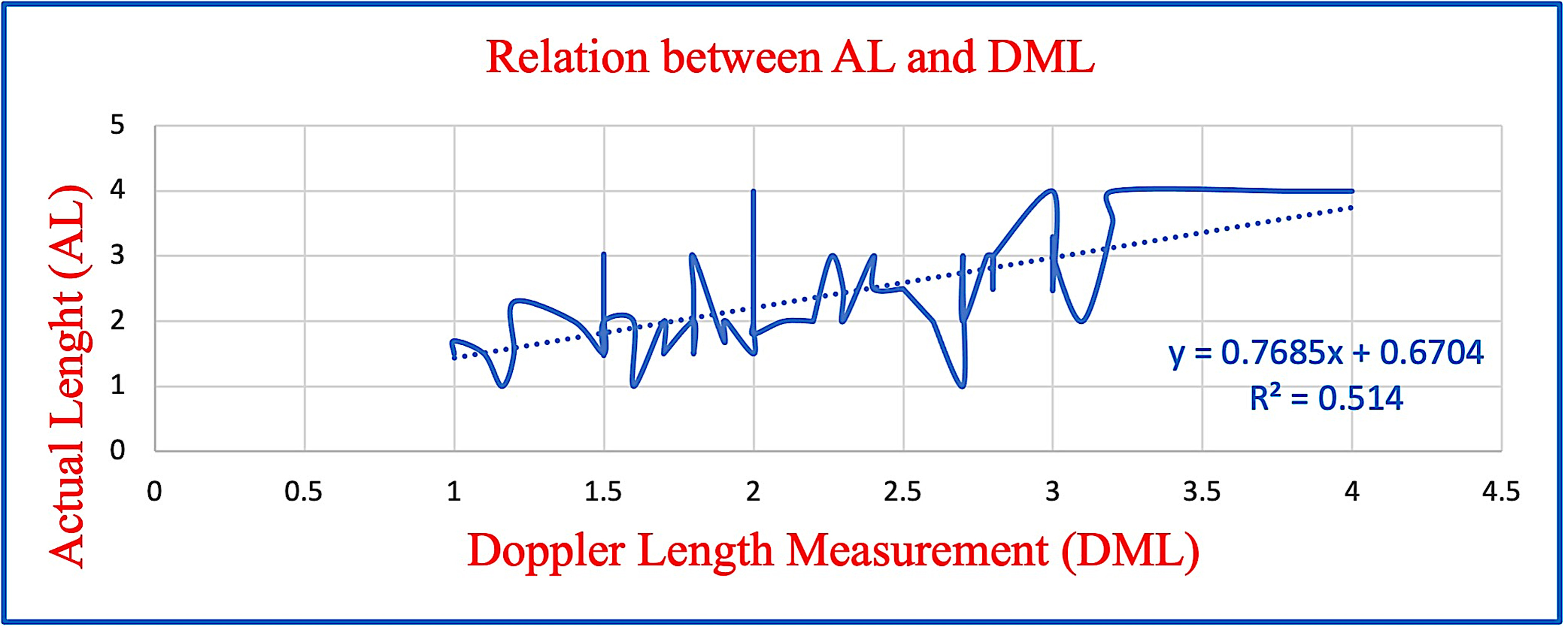
Fig. 3 The actual length (AL) measurement can be predicted by the Doppler measurement length (since they are positively and statistically significantly related) using the following equation: *AL*=0.6704+0.7685·*Doppler*.

It is clear that the AL measurement can be predicted by the DML (since they are positively and statistically significantly related) using the following equation (**Fig. 3**):
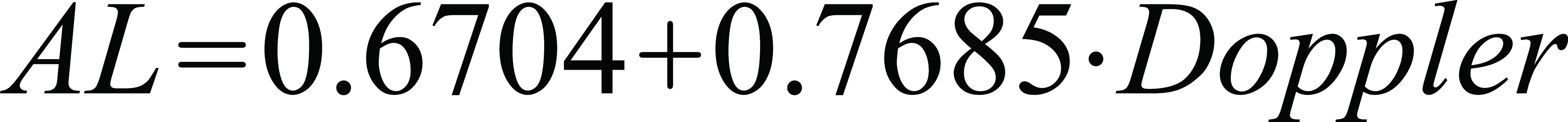


The relation between the AL and DML leads to the following conclusion: for Doppler measurement length shorter than 2.9 cm, the predicted AL is longer than the measured Doppler length. For Doppler measurement length equal or longer than 2.9 cm, the predicted AL is shorter than the measured Doppler length.

2. Multiple linear regression of the AL measurement (dependent variable) on the DML (independent variable), body mass index, smoking status (smoker=1, nonsmoker=0), presence of diabetes (diabetic=1, nondiabetic=0), presence of hypertension, (hypertensive=1, nonhypertensive=0), gender (female=1, male=0), and location of the obstruction (left carotid artery=1, right=0)

None of the risk factors nor the gender or the location of the obstruction have a statistically significant relation to the AL, and the goodness of fit of the model (i.e., its predictive validity) is not improving (adjusted R^2^=0.5078 vs. 0.5080).

Finally, the absolute differences of the three methods (preoperative DML, intraoperative measurement of the atherosclerotic plaque from the carotid bifurcation to the supposed distal end before the division SURG, and postoperative histological measurement HISTO) from the AL were compared with each other using parametric and nonparametric ANOVA. Consequently, the Doppler and histological methods have no difference, while the use of the SURG provides clearly poorer results. Following the three-way comparison, all two-way comparisons were performed to identify the source of the difference (**Table 2**).

**Table 2:** Table 2 Comparison of the mean absolute differences from the actual length (all three pair-wise comparisons)

Comparisons	t-test (p-value)	Signed-rank test (p-value)
DML vs. HISTO	0.4122	0.7653
DML vs. SURG	<0.0001	<0.0001
HISTO vs. SURG	<0.0001	<0.0001

DML: Doppler measurement length; SURG: measurement of the atherosclerotic plaque from the carotid bifurcation to the supposed distal end before division; HISTO: histopathological measurement

## Discussion

Modern surgical treatment of carotid disease includes two methods: open surgery and endovascular surgery. The indications for endovascular therapy are limited, and this subject is not within the scope of discussion of this article.^1)^

ECEA has been validated as a safe and effective surgical treatment for carotid stenosis for the prevention of stroke. It is an anatomical procedure that reduces ischemic and total surgical time, excludes the use of a synthetic implant and the potent associated prosthetic infection, and corrects the kinking, preserving the bifurcation geometry. The technique is associated with low restenosis rates due to the ellipsoid suture line, and the appearance of a false anastomotic aneurysm is almost impossible.^2,7–11)^

Due to the above advantages of the eversion technique, the method appears to be progressively removed from the shadow of classical endarterectomy, although it remains not widely accepted by the vascular surgery community; this may be due to tradition and a habit in performing the standard conventional endarterectomy technique, and the subsequent insufficient imparting of the ECEA technique to younger vascular surgeons. In classical endarterectomy through the elongated arteriotomy, the fixation of the distal residual atheroma through tacking sutures and the use of shunt during carotid clamping are taught with relative ease^10)^; in ECEA, a shunt cannot be easily inserted until eversion is completed, and there may be problems in accessing the distal ICA, if a distal extension of the atheroma has been underestimated before the division of the vessel. In this study, ECEA was performed in all patients without needing to apply shunt.

It is well known that the most important technical problem in carotid endarterectomy is the distal extension of the atherosclerotic plaque, which is up to the skull base.^2,7,12)^ This could be an unpleasant revelation after having chosen to perform, in particular, an eversion endarterectomy and in front of an already divided ICA. At this point of no return, the procedure becomes more complicated, although there are several techniques that provide additional exposure of ICA and extensive exposure to the skull base. These techniques include the division of the digastric, sternocleidomastoid artery, occipital branch of the external carotid artery (ECA), transection of the ansa cervicalis, and transection of the styloid process.^1,13,14)^ In the present study, in some 80 patients, the digastric muscle had to be dissected to facilitate access to the ICA and safely perform ECEA. Thus, it is imperative to know if there is enough distal space for clamping before the division of the artery.

The correct preoperative Doppler ultrasonography and corroborative CTA/MRA imaging studies are the gold standards for the appropriate planning of the ECEA. A technical challenge is the high bifurcation or stenosis extending behind the jaw, increasing the risk of cranial nerve injury. In that case, if preoperative extensive head extension is needed, there are other more specific techniques that allow further exposure of the peripheral ICA, including an extension of the incision anterior to the ear with mobilization of the superficial lobe of the parotid, nasopharyngeal intubation, subluxation of the temporomandibular joint, and an extension of the incision to the skull. Most of these techniques require rigorous preoperative programming with the involvement of other specialties, such as ENT or neurosurgeons.^1,13,15–19)^

In the case that the oblique carotid division had been performed and the atherosclerotic plaque is extended peripherally, without the possibility to further perform ECEA, experienced manipulations are needed to continue the operation, apart for the aforementioned paths; these manipulations include the interposition of a graft with end-to-side anastomosis to the distal CCA and end-to-side or end-to-end anastomosis to the distal ICA with ECA exclusion or bifurcation reconstruction.^1,20–23)^

In this study, we evaluated common practice methods to identify if there is a small group of patients with anatomical contraindications for ECEA, due to extensive distal atheromatic disease; an ideal reliable method helps the surgeon exclude ECEA before surgery or just before the division of the carotid and proceed with the conventional endarterectomy.

We analyzed four measurements of atherosclerotic plaque (two indirect and two direct) at four different time intervals (DML, SURG, AL, and HISTO). Firstly, the preoperative meticulous DML was performed using the same ultrasound device and by the same radiologist, always in the presence of a vascular surgeon, to avoid any deviation from the results. Secondly, in addition to preoperative measurements and observations, intraoperative findings through the surgeon’s senses are also important for assessing the distal extension of the atherosclerotic plaque (measurement with a ruler of the tactile atheromatic plaque, before the vessel division, SURG). Thirdly, the internal carotid plaque was removed en bloc, without fragmentation or significant distortion with minimal manipulation of the specimen; a carotid plaque was obtained immediately after endarterectomy, and its AL was measured using the ruler to avoid the possibility of shrinkage that might be achieved, although no literature related to this exists.^24–26)^ Fourthly, after removal, the section of the plaque was placed in formaldehyde solution for the histological measurement of the length of the internal carotid plaque and its histological examination (HISTO).

ECEA was performed in 80 patients without any intraoperative complication. Also, no pathological neurological findings were observed in any patient, from the intubation phase to the early postoperative period, not even for a period of 30 days or after 3 years. Analyzing the results of our study, we have found that the preoperative method of DML is a useful predictor of the AL of atheroma; the preoperative DML has the smallest mean difference from the AL and is close to the histological result. Ultrasound Doppler measurement is useful for predicting the AL of the atheroma and essentially a key criterion for the preoperative assessment of the distal extension of the atheroma. In fact, a correct estimation of the anticipated AL, following preoperative Doppler studies, as is drawn from our results, is in agreement with the aforementioned equation (*AL*=0.6704+0.7685·*Doppler*).

The intraoperative measurement of the atherosclerotic plaque from the carotid bifurcation to the supposed distal end before the vessel division (SURG) significantly differs from the previous methods, without being a reliable criterion for the application of the eversion endarterectomy method.

Moreover, in this case series of patients, for anatomical reasons, no deviation from the eversion technique was necessary. In all cases, the criteria we had prospectively addressed in this study, such as the preoperative ultrasound measurement (DML) and the intraoperative measurement of the atheroma, confirmed the correct decision to proceed with the ECEA technique.

According to the results of this study, it appears that the peripheral distal extension of the atherosclerotic plaque in the ICA can be successfully treated with eversion endarterectomy, based on the criteria of the preoperative color Doppler ultrasound image, as well as intraoperative tactile estimation. Ultrasound Doppler study is a reliable method for assessing the extent of atheroma and therefore a valid criterion for the application of ECEA technique.

The aim of the study was to identify preoperative, intraoperative, and postoperative studies and measurements as surgical criteria for the implementation of eversion technique, and the goal was achieved. The number of patients included in this study is not too large, but this can be compensated by the rigorous planning of the prospective study and the fact that all patients were operated by the same surgeon and the ultrasound studies were conducted by the same radiology operator. Larger prospective studies are welcome to support our results.

## Conclusion

ECEA offers many advantages and should be the first choice in surgical endarterectomy and in restoring the patency of the ICA, according to the latest guidelines of the European Society of Vascular Surgery (Management of atherosclerotic carotid and vertebral artery disease, 2017 clinical practice guidelines of the ESVS, R: 55). If it is performed by an experienced surgeon, it is safe.^1,12,27)^

According to the present study, a preoperative color Doppler ultrasound image is a reliable preoperative estimator of the peripheral extension of the atherosclerotic plaque in the ICA.
